# Collateral Sensitivity of Parthenolide via NF-κB and HIF-α Inhibition and Epigenetic Changes in Drug-Resistant Cancer Cell Lines

**DOI:** 10.3389/fphar.2019.00542

**Published:** 2019-05-21

**Authors:** Mona Dawood, Edna Ooko, Thomas Efferth

**Affiliations:** Department of Pharmaceutical Biology, Institute of Pharmacy and Biochemistry, Johannes Gutenberg University, Mainz, Germany

**Keywords:** drug resistance, chemotherapy, HDAC, natural products, NF-κB, pharmacogenomics, phytochemicals

## Abstract

Parthenolide (PT) is a sesquiterpene lactone isolated from *Tanacetum parthenium*. In this study, PT showed varying cytotoxic effects against different solid tumor cell lines. HCT116 (p53^+/+^) colon carcinoma cells and their parental HCT116 knockout p53 (p53^-/-^) cell lines showed a resistance degree of 2.36. On the other hand, wild-type U87.MG cells or cells transfected with a deletion-activated *EGFR* cDNA (U87.MGΔEGFR) exhibited slight sensitivity toward PT. Multidrug-resistant MDA-MB-231-BCRP cells were even more sensitive toward PT than sensitive MDA-MB-231-pcDNA cells with a resistance degree of 0.07 (collateral sensitivity). To the best of our knowledge, hypersensitivity (collateral sensitivity) in MDA-MB-231-BCRP cell line is reported in this study for the first time. We attempted to identify the mechanism of collateral sensitivity. Firstly, we found that PT bound to IKK preventing IκBα degradation and eventually inhibition of the nuclear factor kappa B (NF-κB) pathway. Down-regulation of hypoxia inducing factor 1-alpha (HIF-1α) in MDA-MB-231-BCRP resistant cells may be a second mechanism, since it is a target gene of NF-κB. Moreover, PT also showed epigenetic effect by inhibition of HDAC activity as shown using both molecular docking and HDAC activity assay. Based on COMPARE and hierarchical cluster analyses, we found gene expression profiles that predicted sensitivity or resistance of 47 tumor cell lines toward PT. Interestingly, pathway analyses of gene expression profiles revealed NF-κB and HIF signaling as top networks of these genes, cellular functions and canonical pathways influencing the activity of PT against tumor cells. In conclusion, PT exerted profound cytotoxic activity against various cancer cell lines mainly against BCRP-overexpressing tumor cells, suggesting PT as novel candidate for cancer treatment.

## Introduction

Parthenolide (PT) is a sesquiterpene lactone isolated from the Mexican-Indian medicinal plant *Tanacetum parthenium*. It has anti-inflammatory properties and is clinically used for migraine treatment (Murphy et al., 1988; Bork et al., 1997). Moreover, PT is nucleophilic in nature due to its lactone ring and epoxide group. This feature explains, why PT exerts several biological activities, such as anti-cancer activity by inducing extrinsic and intrinsic pathways of apoptosis (Wen et al., 2002; Zhang et al., 2004) without affecting normal cells (Mathema et al., 2012).

In addition, PT had a potent effect on both breast cancer stem cells and breast cancer cells. PT suppressed overexpression of NF-E2-related factor 2 (Nrf2) and its related genes that prevented development of resistance to mitoxantrone and doxorubicin in triple-negative breast cancer (Carlisi et al., 2017). PT inhibited MCF-7 mammosphere formation and MCF-7 xenograft tumor growth as well as elimination of breast cancer stem cells by NF-κB pathway deactivation (Dandawate et al., 2016).

Despite the availability of numerous clinically established standard cytostatic drugs, their effectiveness is largely hampered by the development of resistance and severe side effects (Efferth and Oesch, 2004; Kuczynski et al., 2013; Efferth, 2017). Multidrug resistance (MDR) represents a main reason for chemotherapy failure. Among the most important MDR mechanisms are ATP binding cassette (ABC) proteins expressed on cancer cell membranes (Efferth, 2001; Gillet et al., 2007; Boonyong et al., 2017; Efferth and Volm, 2017; Umsumarng et al., 2017). BCRP belongs to this family of efflux transporters responsible for drug disposition and distribution. BCRP expression is significantly associated with tumor response to chemotherapy and resistance (Faneyte et al., 2002; Wu et al., 2018). Mutations in the EGFR also confer drug resistance in non-small cell lung cancer (NSCLC) and other tumor types (Cortot and Janne, 2014), e.g., the T790M mutation, alternative pathways activation, loss of function of the EGFR-mediated apoptosis pathway (Huang and Fu, 2015). In addition, loss of tumor suppressor function such as p53 mutations also play an important role in drug resistance (Hientz et al., 2017; Heo et al., 2018). P53 plays a key role in the regulation of cell cycle arrest, DNA repair and apoptosis. Hence, it effectively contributes to sensitivity toward anticancer drugs (Lowe et al., 1993; Ferreira et al., 1999). A better understanding of the molecular mechanisms underlying resistance to cancer drugs can assist the design of new therapeutic drugs and the development of better treatment strategies to overcome resistance.

Epigenetics plays an important role in tumorigenesis and cancer development. It covers three different areas: DNA methylation, histone modification and non-coding RNAs (Sharma et al., 2010). Histones have a variety of posttranslational modifications at specific residues to regulate DNA replication, transcription and repair. These modifications include methylation, acetylation, ubiquitylation, sumoylation, and phosphorylation (Kouzarides, 2007). Histone acetyltransferase (HAT) has a major impact on genes expression. HDAC counteract HAT (Li and Seto, 2016). Many cancers exhibit defects in the balance between HAT and HDAC activity, which leads to transcriptional silencing of genes that control differentiation, apoptosis, cell cycle arrest, etc. (Lakshmaiah et al., 2014). Therefore, HDACs became an important target for the development of new anticancer therapy.

The transcription factor nuclear factor kappa B (NF-κB) represents a key player in tumorigenesis, because of its mechanistic link to cell proliferation, survival, apoptosis, and metastasis (Lin and Karin, 2003). NF-κB is inactive in the cytoplasm through its binding to inhibitory proteins (IκB). Upon activation by specific stimuli, IκB is phosphorylated by the IκB kinase (IKK) complex. Then, activated NF-κB translocates to the nucleus and binds specific nucleotide sequences at the promoter region of downstream genes, which mediate the expression of these genes (Baud and Karin, 2009). NF-κB target genes are for instance the anti-apoptotic protein Bcl-2, Bfl-1, TNF-receptor associated factors (TRAFs), Bcl-X_L_, Bcl-w, IAPs, c-FLIP, XIAP and other genes (Pahl, 1999). NF-κB activation is also correlated with the down-regulation of the pro-apoptotic proteins Bax and Bad (Cao et al., 2013). The activity of NF-κB in hematologic malignancies and different solid tumors has been associated with anti-cancer drug resistance (Prasad et al., 2010; Luo et al., 2015). Inhibition of NF-κB signaling represents an effective strategy to improve the effectiveness of conventional anti-tumor drugs (Longley and Johnston, 2005).

In the present study, we investigated the cytotoxic effect of PT toward BCRP-transfected breast cancer cells, EGFR-mutated brain cancer cells, and colon cancer cells with a knockout mutation in the *TP53*. These cells exert resistance to conventional anticancer drugs (Kuete et al., 2016, 2017). Therefore, we were interested to find out, whether these drug-resistant cells would be responsive to PT. To understand the mechanisms of sensitivity or resistance of PT to cancer cells, microarray-based mRNA expression profiles were analyzed. Furthermore, we carried out NF-κB and HIF-α expression analyses and HDAC inhibition assays.

## Materials and Methods

### Cell Lines and Reagents

Cancer cell lines were routinely cultured in DMEM medium supplemented with 10% FBS and 1% penicillin/streptomycin (Invitrogen, Darmstadt, Germany) (Bunz et al., 1998; Efferth et al., 2003).

Wild-type U87.MG cells and its transfected sublime U87.MGΔEGFR carrying an *EGFR* cDNA with a deletion of exons 2–7 were obtained from Dr. W. K. Cavenee (Ludwig Institute for Cancer Research, San Diego, CA, United States) (Huang et al., 1997; Saeed et al., 2014). Breast cancer cells transfected with control vector (MDA-MB-231-pcDNA) or with cDNA encoding the BCRP (MDA-MB-231-BCRP clone 23) were previously reported (Doyle et al., 1998). In addition, colon cancer cell lines HCT-116 (p53^+/+^) and it counterparts knockout clones (p53^-/-^) were kindly gifted by Dr. B. Vogelstein and H. Hermeking (Howard Hughes Medical Institute, Baltimore, MD, United States) (Bunz et al., 1998). The above mentioned resistance cell lines were maintained in 800 ng/ml geneticin (Sigma-Aldrich, Taufkirchen, Germany), in order to maintain the transcript. PT was purchased from Sigma-Aldrich. According to the company instructions, it is HPLC level of more than 98%.

### Cell Growth Inhibition Assay

The cytotoxicity of PT was evaluated using the resazurin (Promega, Mannheim, Germany) reduction assay as previously described (Kuete et al., 2016, 2017). Only viable cells can reduce and convert resazurin to highly fluorescent resorufin, while dead cells cannot convert resazurin dye (O’brien et al., 2000). Based on this principle, tumor cells were treated with different concentrations of PT and incubated for 72 h. An Infinite M2000 Proplate reader (Tecan, Germany) was used to measure the fluorescence using excitation/emission wavelength of 544/590 nm. The 50% inhibition concentrations (IC_50_) were determined using dose response curves of each cell lines using Excel 2013 software (Microsoft, Redmond, WA, United States). The experiments were conducted three times independently with six replicates each.

The tumor cell line panel of the National Cancer Institute (NCI, United States) was treated with PT and subjected to the sulforhodamine B assay (Rubinstein et al., 1990).

### COMPARE and Hierarchical Cluster Analyses

The mRNA microarray data of 47 tumor cell lines of the panel of the National Cancer Institute (NCI), United States were subjected to COMPARE analyses to generate rank-ordered lists of candidate genes related to sensitivity or resistance to cytotoxic test compounds as previously reported (Paull et al., 1989). Every gene was ranked for similarity of its mRNA expression values to the log_10_IC_50_ values of PT, in order to create scale index of correlation coefficients (*R*-values). Hierarchical cluster analysis using the Ward method was applied to classify the objects by calculation of distances based on the closeness between-individual distances, resulting in tree clustering termed dendrogram (Efferth et al., 1997; Scherf et al., 2000).

Hierarchical clustering and dendrogram analyses were piloted using CIM miner software^1^. Importantly, COMPARE analyses and Cluster models have been previously validated for gene expression profiling and for approaching molecular pharmacology of anti-tumor compounds. This method was previously described by us in detail (Dawood et al., 2018).

### BCRP ATPase Activity Assay

A colorimetric ATPase assay was carried out to test the effect of PT on ABC transporters. Membranes with human BCRP were purchased from Corning Life Sciences (NY, United States). The assay was conducted following the manufacturer’s protocol. We previously described the ATPase protocol in details (Ooko et al., 2016; Hamdoun et al., 2017). Briefly, a reaction mixture composed of membranes, PT concentration, MgATP and assay buffer was incubated for 20 min at 37°C. To stop the reaction, 10% SDS was added. Afterward, a color reagent was added to the wells, in order to measure inorganic phosphate using Tecan Reader Infinite m200 Pro. The assay was performed in triplicate. Nunc transparent flat-bottomed plates were used for the measurements. Sulfasalazine was used as positive control.

### Molecular Docking

Molecular docking is a predictive bioinformatical method to evaluate the interaction of ligands with their target proteins. The three-dimensional structure of PT was obtained from the PubChem database using PT smiles (Simplified Molecular Input Line Entry Specification), while the X-ray crystallography-based three-dimensional structures for the desired target proteins were downloaded from the Protein Data Bank^2^; the selected proteins and their PDB ID’s are represented in Table 3.

Molecular docking was performed with AutoDock 4 by means of a Lamarckian algorithm as previously described by our group (Morris et al., 2009; Zeino et al., 2014). Protein structures were initially processed with AutoDock Tools, in order to overcome structural problems due to missing atoms or water. The PDBQT output format was prepared. Then, a grid box was created to direct the docking process. 250 runs and 2,500,000 energy evaluations were set as docking parameters for each round. Visual Molecular Dynamics (VMD) was carried out for visualization of the interaction modes obtained from docking experiments (Zeino et al., 2014; Kadioglu et al., 2015). Vorinostat and triptolide were applied as control inhibitors for HDAC and NF-κB, respectively. The experiments were performed three times and the mean of the lowest binding energies and mean binding energies were taken into account.

### NF-κB Reporter Assay

NF-κB SEAP reporter HEK 293 were purchased from InvivoGen (San Diego, CA, United States), in order to examine the effect of PT on NF-κB. They express the secreted embryonic alkaline phosphatase (SEAP) reporter gene under the regulatory sequence of the NF-κB promoter.

HEK293 cells were culture in appropriate conditions as previously described (Seo et al., 2016; Hamdoun and Efferth, 2017). HEK293 cells were treated with different concentrations of PT followed by 100 ng/mL of tumor necrosis factor (TNF) for 24 h to activate NF-κB. Pre-warmed Quanti-Blue reagent (InvivoGen) was applied to measure SEAP levels using an Infinite M2000 Pro plate reader at 630 nm. Triptolide (1 μM, InvivoGen) was used as positive control. For these experiments, the assay was repeated at least three times.

### Protein Extraction

MDA-MB-231-pcDNA and MDA-MB-231-BCRP cells were seeded in six-well plates and treated with different PT concentrations (5, 10, and 25 μM). After 6 h incubation, cells were washed with PBS and harvested in 1.5 ml Eppendorf tubes. The total protein fraction was extracted using M-PER^®^ Mammalian Protein Extraction Reagent (Thermo Fisher Scientific, Germany) with protease inhibitor (1:100) and shaken for 30 min at 4°C. Then, cell lysates were centrifuged at 14,000 × *g* for 15 min at 4°C. The supernatants were collected in clean tubes. Protein quantity and quality were measured by Nano-Drop 1000 (Thermo Fisher Scientific) (Hamdoun and Efferth, 2017).

### SDS-PAGE and Western Blot Analysis

Thirty mg/ml were taken from the protein fraction, and SDS-loading dye was added following by heating at 95°C for 10 min. After the denaturation process, proteins were loaded onto 10% SDS-polyacrylamide gels. A Western blotting apparatus was used to transfer proteins on a PVDF membrane (Roti^®^ PVDF, pore size 0.45 μm, Carl Roth GmbH, Karlsruhe, Germany). The membrane was blocked using 5% BSA/TBS-T and then incubated with primary antibodies against NF-κB p65 (D14E12), IκB (44D4), HIFα (D2U3T), or β-actin (13E5) overnight at a dilution of 1:1000. HRP-linked secondary anti-rabbit antibody (1:2000) was then added and incubated for 1 h. Both primary and secondary antibodies were purchased from Cell Signaling (Frankfurt am Main, Germany). Luminata Classico HRP Western Blot substrate (Merck Millipore, Schwalbach, Germany) was used for the detection step and membranes was visualized with aid of Alpha Innotech FluorChem Q system (Biozym, Oldendorf, Germany) (Saeed et al., 2015; Zhao et al., 2015).

### HDAC Activity Assay

Histone deacetylase activity assay kit (free cell assay) were purchased from Abcam (Cambridge, CB4 0FL, United Kingdom). The assay was performed following the manufacturer’s instructions to measure the activity of HDAC in the presence or absence of PT. The assay measures the activity of crude HDAC by the basic principle of changing an HDAC reaction into peptidase activity. After incubation with the compounds for at least 20 min at room temperature, fluorescence intensity was read using Infinite M2000^TM^ Pro plate reader (Tecan) at Ex/Em = 355 nm/460 nm. DMSO was used as negative control, while vorinostat and trichostatin were used as positive controls (Xie et al., 2014). The experiments were repeated twice.

### Ingenuity Pathway Analysis

A number of software programs and bioinformatical tools have been developed to identify the relationship between set of proteins and whether they contribute to specific pathways. Examples are open access programs such as Kyoto Encyclopedia of Genes and Genomes (KEGG) (Ogata et al., 1999), WikiPathways and Reactome (Vastrik et al., 2007), while other programs such as Pathway Studio and to Ingenuity Pathway Analysis (IPA) are commercially available (Panguluri et al., 2013).

In the present manuscript, genes described and identified via compare analysis as factors determining cellular responsiveness to PT were subjected to IPA (Qiagen Bioinformatics, Redwood City, CA, United States). Prior to the analysis, genes were uploaded to IPA in Excel format. Core analyses were carried out to identify canonical pathways, diseases and functions, and relevant networks as described (Dawood et al., 2018).

### Statistical Analysis

Pearson’s correlation test was applied to correlate microarray-based mRNA expression of candidate genes with the IC_50_ values for PT. Hierarchical cluster analysis using Ward’s method (WinSTAT program, Kalmia, Cambridge, MA, United States) was also used. Student’s *t*-test using Microsoft Excel 2016 was performed to calculate the statistical significance of PT effect on NF-κB pathways, HDAC activity, ATPase activity and HIF-α expression. *P*-values of less than 0.05 were considered as significant. All data represent mean values ± SD of three independent experiments.

## Results

**FIGURE 1:**
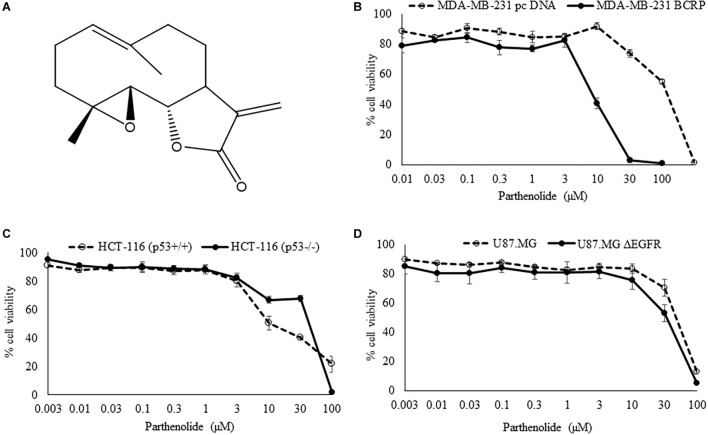
**(A)** Chemical structure of parthenolide. **(B–D)** Cytotoxicity of parthenolide toward sensitive and drug-resistant tumor cell lines as measured by the resazurin reduction assay. Experiments have been performed as triplicates.

We tested PT in cell models expressing three different drug resistance mechanisms (mutant EGFR, knockout p53, overexpression of BCRP) using resazurin reduction assays. The degree of resistance was calculated by dividing the IC_50_ of HCT116 knockout p53 cells (p53^-/-^) by the IC_50_ of parental wild-type cells HCT116 (p53^+/+^). Only a weak cross-resistance of the knockout HCT116 (p53^-/-^) cells was observed (2.36-fold). In addition, U87.MG wild-type cells and their counterpart glioblastoma cells transfected with a deletion-activated *EGFR* cDNA (U87.MG.ΔEGFR) were investigated. U87.MG.ΔEGFR cells exhibited a slight sensitivity toward PT with an IC_50_ value of 32.7 ± 3.8 μM, which was lower than the IC_50_ value of wild-type U87.MG cells (46.0 ± 3.8 μM) (Table 1). Concerning the ABC-transporter BCRP/ABCG2, it was an unexpected, but pleasing result that the multidrug-resistant MDA-MB-231-BCRP cell line was considerably more sensitive toward PT than sensitive MDA-MB-231-pcDNA cells with a degree of resistance of 0.07 (Table 1). The dose response curves are illustrated in Figure 1.

**Table 1:** Cytotoxic effect of parthenolide toward different cancer cell lines.

Cell lines	Parthenolide	
	
	IC_50_ (μM)	Degree of resistance
HCT116 p53^+/+^	17.6 ± 1.8	2.36
HCT116 p53^-/-^	41.6 ± 1.2	
U87.MG	46.0 ± 3.8	0.77
U87.MG ΔEGFR	32.7 ± 3.8	
MDA-MB-231 pc DNA	115.8 ± 2.3	0.07
MDA-MB-231 BCRP	08.5 ± 1.3	


*The IC_*50*_ values were shown as mean values ± SD of triplicate experiments with each six parallel measurements.*

### COMPARE and Hierarchical Cluster Analyses

The transcriptome-wide mRNA expression of 47 cell lines from different tumor types were correlated to the log_10_IC_50_ values for PT. This bioinformatical COMPARE analysis based on Pearson’s rank correlation test was applied to identify novel putative molecular factors associated with cellular response to PT. The top 20 genes positively correlating with log_10_IC_50_ values more than *R* = 0.50 and the top 20 genes negatively correlating with log_10_IC_50_ values less than *R* = -0.50 negative are summarized in Table 2 together with their specific cellular functions.

**Table 2:** Correlation coefficients of mRNA expression to log_10_IC_50_ values obtained using COMPARE analyses for 47 NCI cancer cell lines and genes function obtained from gene cards and gene atlas databases.

Gene Symbol	Gene name	Gene function
RRP1B	Ribosomal RNA processing 1 homolog B (*S. cerevisiae*)	Metastasis, modulator of transcription and chromatin, role in the regulation of gene, induction of apoptosis.
EMG1	EMG1 nucleolar protein homolog (*S. cerevisiae*)	Ribosomal subunit biogenesis.
NPM3	Nucleophosmin/nucleoplasmin 3	Inhibits histone assembly activity of NPM1 and dramatically enhances transcription. This protein likely functions as a molecular chaperone in the cell nucleus.
COIL	Coilin	
ARHGAP4	Rho GTPase activating protein 4	Inhibition of stress fiber organization. Role in lymphocyte differentiation. Cell organization/biogenesis.
RPL18A	Ribosomal protein L18a	Protein translation and synthesis.
PDCD11	Programmed cell death 11	NF-κB (NFKB1; 164011)-binding protein. Required for rRNA maturation and generation of 18S rRNA.
IL27RA	Interleukin 27 receptor, alpha	Receptor for IL27. Can trigger signaling in T cells, B cells, and myeloid cells.
POLR3C	Polymerase (RNA) III (DNA directed) polypeptide C (62 kD)	Nucleotide transcription regulation.
RPL36A	Transcribed locus, strongly similar to NP_775369.1 60S ribosomal protein L36a	Ribosomal protein. Role in tumor cell proliferation.
NOLC1	Nucleolar and coiled-body phosphoprotein 1	Cell cycle, division, mitosis.
RPL17	Ribosomal protein L17	Signaling function.
E2F3	E2F transcription factor 3	Transcription repression in quiescent cell by interaction with histone deacetylase. Cell cycle.
RGS19	Regulator of G-protein signaling 19	Inhibiting signal transduction.
GNL2	Guanine nucleotide binding protein-like 2	GTPase that associates with pre-60S ribosomal subunits in the nucleolus and is required for their nuclear export and maturation.
RBM34	RNA binding motif protein 34	RNA recognition motif protein.
PPRC1	Peroxisome proliferator-activated receptor gamma, coactivator-related 1	Involved in mitochondrial proliferation.
NACA	Nascent polypeptide-associated complex alpha subunit	Transcriptional co-activator.
FAM216A	Family with sequence similarity 216 member A	Unknown.
NVL	Nuclear VCP-like	ATP-dependent zinc metallopeptidase. Role in ribosome biosynthesis. Essential for telomerase biogenesis.
CLPTM1	Cleft lip and palate associated transmembrane protein 1	Function in developmental processes.
PTPRK	Protein tyrosine phosphatase, receptor type, K	Regulation of processes involving cell contact and adhesion such as growth control, tumor invasion. and metastasis.
CRELD1	Cysteine-rich with EGF-like domains 1	Role in valvuloseptal morphogenesis.
CTNND1	Catenin (cadherin-associated protein), delta 1 RNA	Role in cell structure and adhesion.
PDLIM5	PDZ and LIM domain 5	Role in cytoskeleton organization, cell lineage specification, organ development, and oncogenesis. Actin-associated protein acting as a cytoplasmic retention factor for ID2.
ZBTB20	Zinc finger and BTB domain containing 20	Transcription factor involved in hematopoiesis, oncogenesis, and immune responses.
ALDH3A2	Aldehyde dehydrogenase 3 family, member A2	Catalyzing the oxidation of medium-chain (fatty) aliphatic and aromatic aldehydes to fatty acids. Detoxification of aldehydes generated by alcohol metabolism and lipid peroxidation.
IGFBP4	Insulin-like growth factor binding protein 4	Alters the interaction of IGFs with cell surface receptors.
MEGF8	Multiple EGF-like-domains 8	Intracellular trafficking.
PALLD	Palladin, cytoskeletal associated protein	Targeting ACTN to specific subcellular foci.
SIM2	Single-minded homolog 2 (*Drosophila*)	Role in the development of central nervous system.
ECE1	Endothelin converting enzyme 1	Poteolytic processing of endothelin precursors to biologically active peptides.
MYRF	Myelin regulatory factor	Role for the generation of mature myelin-gene-expressing oligodendrocytes within the CNS.
SNAP25	Synaptosomal-associated protein, 25 kDa	Role in the synaptic function of specific neuronal systems.
HIF1A	Hypoxia inducible factor 1, α subunit (basic helix-loop-helix transcription factor)	Master regulator of cellular and systemic homeostatic response to hypoxia by activating transcription of many genes.
B4GALT4	UDP-Gal:β GlcNAc β 1,4-galactosyltransferase, polypeptide 4	Role in glycosphingolipid biosynthesis.
ANXA2	Annexin A2	Role in the regulation of cellular growth and in signal transduction pathways.
ABCC3	ATP-binding cassette, sub-family C (CFTR/MRP), member 3	ABC transporter, traffic ATPase. Role in the transport of biliary and intestinal excretion of organic anions.
ANXA2P3	Annexin A2 pseudogene 3	Unknown.
RBP4	Retinol binding protein 4, plasma	Delivery of retinol from the liver stores to peripheral tissues.


**FIGURE 2:**
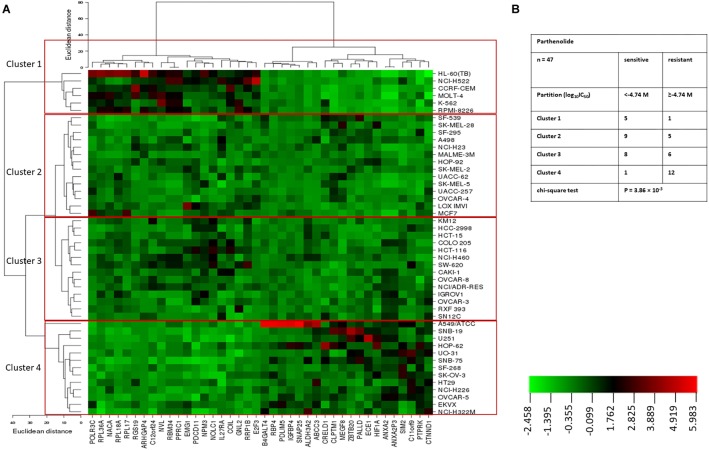
**(A)** Heat map obtained by hierarchical cluster analysis of transcriptome-wide expression profiling of 47 NCI tumor cell lines correlating to sensitivity and resistance toward parthenolide. **(B)** Clusters of NCI tumor cell lines gained by hierarchical cluster analyses for parthenolide. The median log_10_IC_50_ value (M) for parthenolide was used as cut-off to classify tumor cell lines as being “sensitive” or “resistant.”

Hierarchical cluster analysis (cluster image mapping) was conducted for these selected genes. The examined 47 NCI cell lines are depicted on the right side of the heat map (dendrogram), while the 40 genes are placed on the bottom of the heat map (Figure 2A). The cell lines can be divided into four major clusters. The first cluster contained six cell lines, the second and third clusters contained each 14 cell lines, and the fourth cluster consisted of another 13 tumor cell lines. Using the chi-square test, we investigated whether the scattering of these cell lines being sensitive or resistant toward PT was significantly different between these four clusters. We found a *p*-value of 3.86 × 10^-3^ (Figure 2B), indicating that sensitivity or resistance of tumor cell lines to PT was predictable by using this gene expression profile. The identified genes belong to different functional classes such as cell cycle and growth (*E2F3*, *NOLC1*, and *PTPRK*), signal transduction (*ANXA2*, *IGFBP4*, *IL27RA*, *RGS19*, and *RPL17*), transcription and translation (*HIF1A*, *NACA*, *NVL*, *EMG1*, *POLR3C*, and *RPL17*), development and differentiation (*ARHGAP4*, *CLPTM1*, and *PDLIM5*), and apoptosis (*RRP1B*, *HIF1A*, etc.).

### ATPase Activation of BCRP Transporter

**FIGURE 3:**
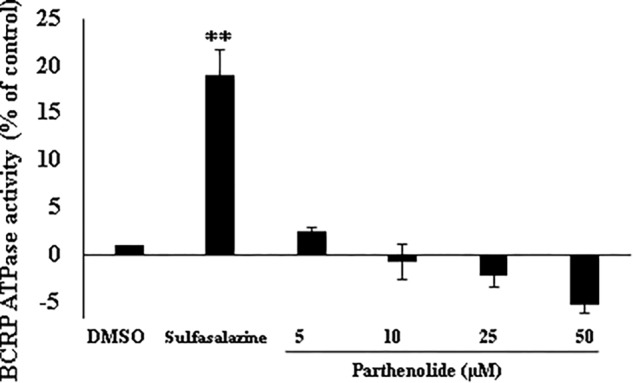
Effect of parthenolide on ATPase activity of BCRP. BCRP-expressing membranes were used to evaluate the inorganic phosphate release from ATP as indicator of ABCG2 transporter activity. Four different concentration were used (5, 10, 25, and 50 μM), and sulfasalazine was used as positive control (known ATPase activator of BCRP transporter). ATPase activity (nmol/min/mg protein) was measured as compared to DMSO control. Asterisks (^∗∗^) indicates the statistical significant induction of ATPase (*p* < 0.01) in compared to DMSO-treated control cells.

To understand why BCRP-overexpressing cells revealed collateral sensitivity to PT, we evaluated the effect of PT on the ATPase activity of BCRP. Human BCRP spotted onto membranes was used to conduct ATPase assays with or without PT treatment. Sulfasalazine, which is known to activate ATPase activity of the BCRP transporter served as positive control. In contrast to sulfasalazine, PT showed statistically insignificant effect on ATPase activity of BCRP in a dose-dependent manner (Figure 3).

### *In silico* Binding of PT to HDAC and NF-κB Proteins

**FIGURE 4:**
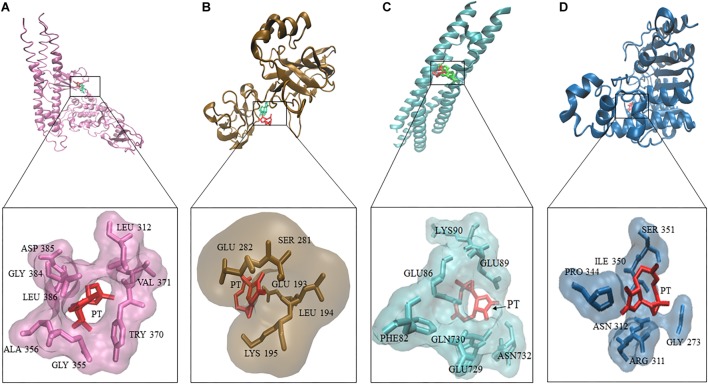
Molecular docking of parthenolide and known inhibitors of the NF-κB pathway and HDAC2. Macromolecules have been represented in new cartoon format, while PT was represented in red. The known NF-κB inhibitor triptolide is shown in green using Visual Molecular Dynamics (VMD) software. **(A)** Docking poses of the IKK pharmacophore (PDB code: 3R2F) in pink color. **(B)** Docking poses of the pharmacophore of NF-κB DNA complex (PDB code: Ivkx) in brown color. **(C)** Docking poses of the pharmacophore of IKK nemo (PDB code: 3BRT) in green. **(D)** Docking poses of the HDAC2 pharmacophore (PDB code: 5IWG) in blue.

Three deregulated genes (E2F3, HIF-α, BCRP) identified by COMPARE analysis in the NCI cell line panel were previously described as downstream target genes of NF-κB (Cheng et al., 2003; Viturro et al., 2006; Belaiba et al., 2007; Bonello et al., 2007). Based on this finding, we performed molecular docking of PT with IκB kinase, IκB kinase-NEMO complex and NF-κB-DNA (p65/p50) complex (Figure 4). PT strongly bound with a binding energy of -8.08 ± < 0.001 kcal/mol to IκK-NEMO and to NF-κB (RelB/p52) with -7.54 ± < 0.001 kcal/mol. Table 3 shows the lowest binding energies for the three proteins and the amino acids residues involved in the interaction with PT. Bold amino acids presented the residues that are involved in hydrogen bond interaction with the compounds. PT showed comparable binding energies to the known NF-κB inhibitor triptolide and with even lower binding energies than triptolide to IκB kinase. Both compounds docked to the same binding site (Figure 4).

**Table 3:** Molecular docking results of parthenolide and known inhibitors of NF-κB and HDAC2.

	Mean binding energy (kcal/mol)	pKi (μM)	Pharmacophore	No. of H bond
**IκK**				
Parthenolide	-6.52	16.53 ± 0.011	GLY384, ALA356, GLY355, LEU386, ASP385, LEU312, TRY370, VAL371, ILE370	0
Triptolide	-5.46	98.98 ± 0.18	GLN355, **ASP358**, GLY384, ILE372, VAL371, LEU312, **LEU386**, TRY370	2
**IκK-NEMO**				
Parthenolide	-8.08	1.19	MET734, GLU89, GLN730, GLN86, PHE82, ASN732, GLU729, LYS90, GLU89, GLN86, **ARG87**	1
Triptolide	-9.59 ± 0.005	0.99 ± 0.002	MET734, GLN86, **GLU89**, LEU93, GLN730, GLN86, ARG89, LYS90, GLN730, GLU89	1
**NF-κB-DNA (p65/p50)**				
Parthenolide	-5.12	177.46 ± 0.16	SER281, **GLU282**, LEU194, **LYS195**, GLU193	2
Triptolide	-5.17 ± 0.12	164 ± 31.5	**ARG30**, GLU193, GLU279, LEU280, SER281, GLU282, PRO283, **GLN271**	2
**HDAC**				
*Parthenolide*	-6.59 ± 0.17	15.14 ± 4.57	Asp351, Arg312, Ile350, Ser351, **Asn312**, **Arg311**, Gly273, Pro344	2
Vorinostat	-8.03 ± 0.21	1.36 ± 0.44	Phe210, Tyr209, **His183**, His146, **Gly154**, His145, Met35, Cys156, Gly306, Leu144, Gly143, Try306	2


*Binding energies and predicted inhibition constants (pKi) are represented as mean ± SD. Amino acids in bold are involved in hydrogen bonding.*

In addition, we performed molecular docking for PT against HDAC. Docking experiments revealed that PT bound to HDAC with a binding energy of -6.52 ± < 0.001 kcal/mol, remarkably, this interaction was lower compared to vorinostat a well-known HDAC inhibitor (Figure 4).

### PT Inhibit NF-κB Pathway

The NF-κB pathway plays a vital role in the activation of genes associated with cell proliferation, angiogenesis, metastasis and suppression of apoptosis. Thereby, this pathway promotes oncogenesis (Xia et al., 2014). It also induces drug resistance in cancer cells (Ahmed et al., 2013).

**FIGURE 5:**
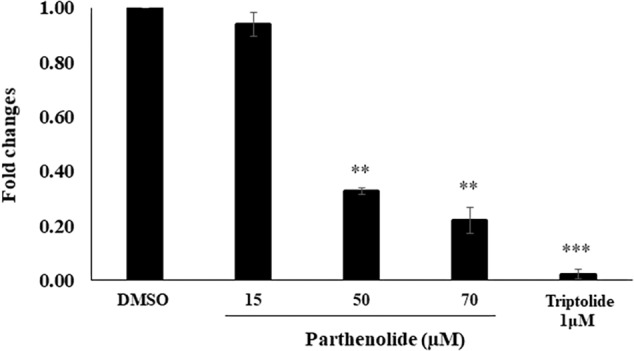
Inhibition of NF-κB activity using HEK-Blue^TM^ cells. Three different parthenolide concentrations (15, 50, and 70 μM) and 1 μM triptolide (TP) were used. The quantification was carried out after 24 h incubation. The results are shown as mean values ± SD of three independent experiments. Asterisks (^∗∗^) indicates statistical significant inhibition (*p* < 0.01) while three asterisks (^∗∗∗^) indicate statistical significant (*p* < 0.001) compared to DMSO-treated control cells.

To confirm the molecular docking of PT to NF-κB, we investigated the inhibitory effect of PT toward NF-κB using a SEAP-driven NF-κB reporter cell line. The HEK Blue Null 1 cells were treated with different concentrations of PT or triptolide as well-known NF-κB inhibitor (Yinjun et al., 2005). Cells treated with DMSO served as negative control. As shown in Figure 5, PT significantly inhibited NF-κB activity in a dose-dependent manner.

### Protein Expression Analysis

**FIGURE 6:**
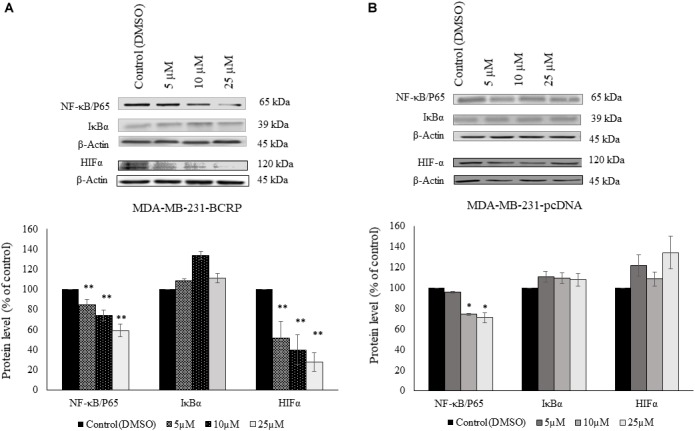
Western blot analysis of NF-κB, IκB, and HIF-1α in parthenolide-treated multidrug-resistant MDA-MB-231-BCRP and sensitive MDA-MB-231-pcDNA3 cell lines **(A)** represents protein analysis for MDA-MB-231-BCRP while **(B)** represents protein analysis for MDA-MB-231-pcDNA. Cells were incubated with 5, 10, and 25 μM parthenolide and DMSO as negative control for 6 h. Then, total protein was extracted and Western blotting was performed. The chart shows the change in the protein expression after normalization to β-actin as mean ± SD for two independent experiments. Asterisk (^∗^) indicates statistical significant inhibition (*p* < 0.05) while two asterisks (^∗∗^) indicate statistical significant (*p* < 0.01) compared to DMSO-treated control cells.

Western blot analysis revealed that PT inhibited NF-κB and HIF-α expression in a dose-dependent manner. Assuming that this may explain the collateral sensitivity of the BCRP overexpressing cells to PT, three different concentrations of PT were applied (5, 10, and 25 μM). Figure 6 showed that NF-κB and HIF-α expression was statistically significant downregulated in multidrug-resistant MDA-MB-231-BCRP cells in comparison to their sensitive counterpart, MDA-MB-231-pcDNA.

### HDAC Activity Assay

**FIGURE 7:**
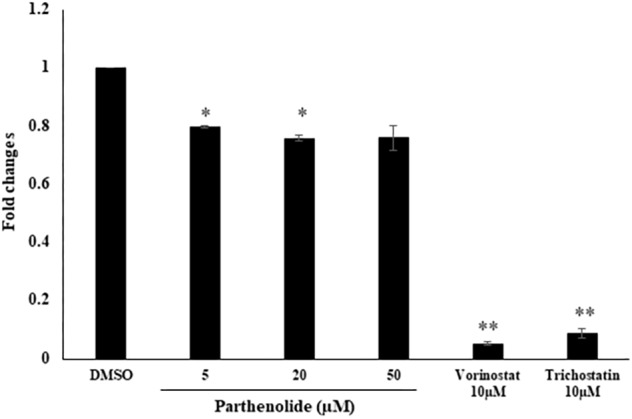
Determination of histone deacetylase (HDAC) activity upon treatment with three parthenolide concentrations. The known HDAC inhibitors vorinostat and trichostatin were used as control compounds at a concentration of each 10 μM. Asterisk (^∗^) indicates statistical significant inhibition (*p* < 0.05) while two asterisks (^∗∗^) indicate statistical significant (*p* < 0.01) compared to DMSO-treated control cells.

To confirm the data obtained from molecular docking of PT to HDAC, we investigated whether PT may possess HDAC inhibitory activity using free cell assay. PT significantly inhibited nuclear HDAC activity at 5 and 20 μM. The known inhibitors vorinostat and trichostatin, which served as positive controls, also significantly inhibited nuclear HDAC (Figure 7).

### Bioinformatic Analysis

**FIGURE 8:**
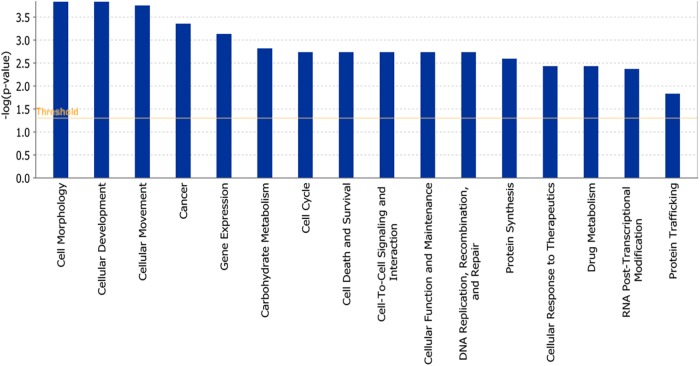
Biological functions affected by parthenolide as determined by mRNA microarray hybridization and Ingenuity Pathway Analysis.

**FIGURE 9:**
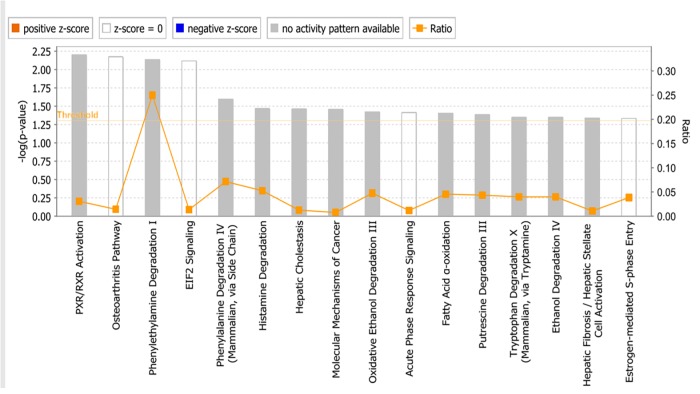
Canonical pathways identified using Ingenuity Pathway Analysis. *P*-values were determined by Fisher’s right tailed exact test. *Y*-axis of the bar showed –log (*p*-value). The different color bars represent the pathway status orange bars are active predicted pathways while blue bars are the inhibited one. In this chart only significant results was represented. Gray bars showed pathways, where no prediction can be applied.

**FIGURE 10:**
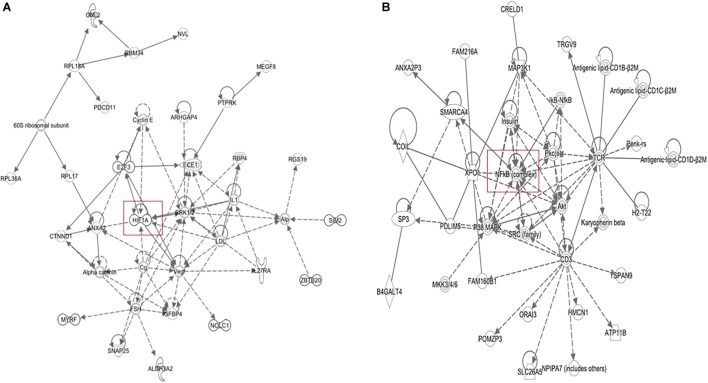
IPA network analyses of the 40 deregulated genes that we obtained from COMPARE analysis (see Table 2) revealed the reconstruction of **(A)** NF-κB network pathway and **(B)** HIF-1α network.

**FIGURE 11:**
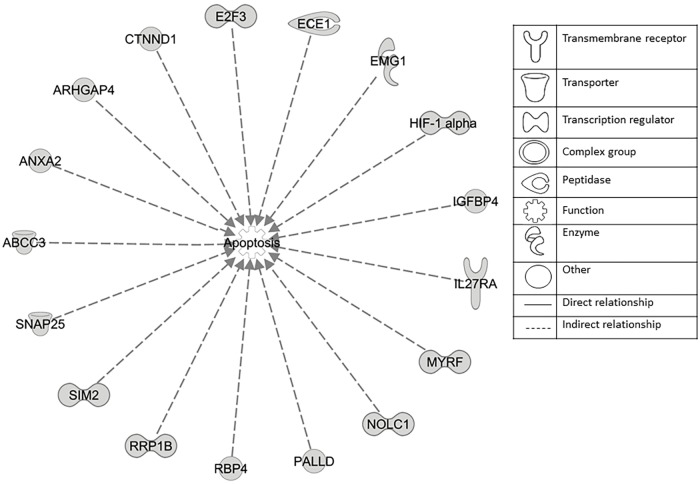
Set of 17 genes involved in apoptosis regulation as identified by COMPARE analysis and IPA analyses.

The microarray data of the COMPARE analysis were subjected to IPA. These genes contributed to important cellular functions and diseases, e.g., cell morphology, cellular development, cellular growth and proliferation, cellular movement, cell death, carbohydrate metabolism, cancer, etc. (Figure 8). On the other hand, the IPA analysis showed important canonical pathways, such as PXR/RXR activation, osteoarthritis pathway, phenylethylamine degradation, EIF2 signaling, phenylalanine degradation, etc. (Figure 9). Interestingly, IPA network analysis showed the NF-κB and HIF-α pathways (Figure 10) as well as apoptosis genes (Figure 11).

## Discussion

In the present study, we undertook an attempt to better understand the multi-target function of PT in the context of drug resistance to established cancer drug. Therefore, we tested three drug resistance phenotypes; EGFR-mutated brain cancer cells (as example of an oncogene), knockout p53 colon cancer cells (as an example of a tumor suppressor gene) and BCRP-transfected breast cancer cells (as an example of a multidrug resistance-mediating ABC transporter). Interestingly, PT showed not only profound cytotoxicity toward these drug-resistant cell lines, but also toward a panel of 47 cell lines of different tumor types. To the best of our knowledge, the collateral sensitivity of BRCP-overexpressing tumor cells to PT is reported here for the first time. Several other studies showed the potential of PT to combat other drug resistance phenotypes. Carlisi et al. (2015) showed that PT prevented drug resistance to mitoxantrone and doxorubicin in MDA-MB231 cells. In another study by Li et al. (2018), PT reversed drug-resistance of human cisplatin-resistant gastric carcinoma cells (SGC-7901/DDP) cells by inhibiting the signal transducer and activator of transcription 3 (STAT3) signaling pathway, increasing the expression of BAX and p53, cleaving caspase-3 and caspase-9, and decreasing Bcl-2 and Bcl-xL expression levels. On the other hand, it also affected cell cycle through increasing cyclin-dependent kinase inhibitor 1 expression and decreasing cyclin D1 expression (Li et al., 2018).

The cytotoxicity of PT found in our analysis has been confirmed by other investigations. PT caused cells death in SW620 cells by inhibiting migration/invasion proteins such as E-cadherin, β-catenin, vimentin, Snail, cyclooxygenase-2, matrix metalloproteinase-2 (MMP-2), and MMP-9 as well as by activating caspase-3 (Liu et al., 2017). It also showed growth inhibitory effects of human cervical cancer (SiHa), breast cancer (MCF-7) cell lines (Al-Fatlawi et al., 2015), non-small cell lung cancer (GLC-82) (Lin et al., 2017), immortalized keratinocytes HaCaT and melanoma cells A375 (George et al., 2016).

Several mechanisms have been proposed to explain collateral sensitivity in ABC membrane transporter-expressing cells. From P-glycoprotein (ABCB1/MDR1), it is known that collateral sensitivity is caused by strong binding of the drug to the ABC transporter, which leads to ATP hydrolysis by the drug pump. Since the drug is not pumped out, another ATP is cleaved – again without success. The futile cycling of ATP hydrolysis leads to preferential ATP depletion and ultimately preferential death of P-glycoprotein expressing cells compared to P-glycoprotein-negative sensitive wild-type cells. Therefore, we tested the ATPase activity of BCRP (ABCG2) after treatment with PT. However, ATPase activity was not changed upon PT treatment of MDA-MB-231 BCRP transfected cells. This indicates that the most common mechanism of collateral sensitivity in P-glycoprotein-expressing cells did not apply to BCRP-expressing MDA-MB-231-BCRP cells used in the present investigation.

To further study the collateral sensitivity of MDA-MB-231-BCRP cells to PT, we focused on NF-κB (Bork et al., 1997; Hayashi et al., 2010), which represents an important target for cancer therapy with more than 700 identified as NF-κB inhibitors, including PT (Gupta et al., 2010). Our molecular docking results showed that PT bound to Iκk with high binding energy, indicating that IκBα phosphorylation may be inhibited. This may results in decreased NF-κB p65 expression via blocking the phosphorylation and degradation of inhibitor of κB-α (IκBα) (Baud and Karin, 2009). We validated the bioinformatical docking using NF-κB reporter assays and Western blotting. Statistically, we found that NF-κB in MDA-MB-231-BCRP was more inhibited than in the sensitive MDA-MB-231 cells.

PT inhibited nuclear factor-κB (NF-κB) signaling (Bork et al., 1997; Hehner et al., 1998; Kishida et al., 2007; Oka et al., 2007). It promotes apoptotic mediated cell death and inhibits NF-κB through the IkB kinase inhibition and/or direct modification of p65 protein (Liu et al., 2010; Kanthan et al., 2012). Transcription of pro-apoptotic genes was suppressed as a result of NF-κB and STAT-inhibition by PT. NF-κB phosphorylation was downregulated in gastric cancer cell lines (MKN-28, MKN-45, and MKN-74) upon PT treatment (Sohma et al., 2011). PT inhibited Iκk resulting in IκBα degradation and subsequent NF-κB pathway inactivation (Hehner et al., 1998; Hehner et al., 1999; Saadane et al., 2007). PT treatment induced apoptosis by inhibition of NF-κB in colitis-associated colon cancer (Kim et al., 2015). Wang et al. (2010) identified a NF-κB consensus binding site within the *BCRP* promoter and validated this bioinformatical result using EMSA assay in MCF-7 cells. This study further supports our findings that PT preferentially inhibited NF-κB in BCRP overexpressing cells leading to collateral sensitivity.

In addition to apoptosis induction through NF-κB, HIF-α is also related to drug resistance, and it is a target of NF-κB too (Gorlach and Bonello, 2008; Xia et al., 2018). Our findings showed that PT inhibited HIF-α in multidrug-resistant BCRP-expressing cells more than in their sensitive counterpart. The preferential inhibition of HIF-α expression by PT in MDA-MB-231-BCRP may, therefore represent a second mechanism of collateral sensitivity.

Solid tumors frequently contain hypoxic regions, as they grow more rapidly than blood supply can follow. In order to overcome this problem, tumor cells activate a cellular hypoxia program making them not only resistant to low oxygen supply but also against chemotherapy. Several studies indicated that HIF-α expression is vital for tumor survival (El Guerrab et al., 2017). Therefore, it represents an attractive target for cancer therapy (Koh et al., 2010). HIF-α expression was significantly correlated with P-glycoprotein expression. Hypoxia diminishes sensitivity to chemotherapy drugs by cell cycle arrest, inhibition of apoptosis, lowering pH value, induction of distant metastasis and cellular metabolism alteration (Mahoney et al., 2003; Xu et al., 2017; Xia et al., 2018). Several studies showed that PT inhibited HIF-1α. PT also suppressed the epithelial-mesenchymal transition of metastasizing tumors (Kim et al., 2017). Furthermore, PT significantly inhibited HIF-α activity and angiogenesis through inhibition of the NF-κB pathway.

Epigenetics is the process of inherited alterations in gene expression without change in DNA sequence (Lakshmaiah et al., 2014; Dawood and Efferth, 2015). HAT that occur at the lysine residue of histone proteins activates the transcription of the genes. This process is carried out by histone acetyl transferase (HAT), while HDAC function antagonistically by removing the modification and lead to the negative result of gene transcription (Bannister and Kouzarides, 2011). HDACs exhibit a pro-oncogenic effect though transcriptional inactivation of genes, which are involved in cell differentiation, apoptosis and cell cycle arrest. Therefore, HDAC inhibition is an attractive target for cancer therapy. HDAC inhibitors become a novel and promising class of anti-cancer drugs. They have complex effects on cellular processes (cell cycle arrest, inhibition of DNA repair, induction of apoptosis) by activating transcription of tumor suppresser genes. Vorinostat and romidepsin are Food and Drug Administration-approved HDAC inhibitors for treatment of T-cell lymphoma (Giannini et al., 2012; Khan and La Thangue, 2012). However, they have the disadvantage that tumor cells frequently develop resistance to these drugs. Therefore, novel HDAC inhibitors with improved features are required.

In our experiments, PT significantly inhibited HDAC activity at concentrations of 5 and 20 μM in comparison to DMSO-treated control cells. To the best of our knowledge, this is the first time to report that PT inhibits HDAC activity. The combination of PT and the pan-HDAC inhibitors vorinostat or LBH589 blocked phosphorylation/activation of IKK and RelA/p65 and activation of JNK1 in human acute myeloid leukemia cells. Interestingly, PT increased HDAC inhibitor-mediated apoptosis in hematopoietic cells through NF-κB inhibition (Dai et al., 2010). Co-administration of PT and HDAC inhibitors caused depletion of glutathione (GSH), release of cytochrome c, caspase 3 activation and apoptosis in MDA-MB-231 breast cancer cells (Carlisi et al., 2015). Activation of IKK2 promoted HDAC1 protein depletion (Gopal and Van Dyke, 2006; Vashisht Gopal et al., 2006). On the other hand, PT caused HDAC1 depletion by the DNA-damage-transducer ataxia telangiectasia mutated protein (Gopal et al., 2007).

Recently, several investigations reported that HDAC inhibitors reduced HIF function in tumor cells (Kim et al., 2001; Jeong et al., 2002; Mie Lee et al., 2003). HDAC stabilized HIF-α by decreasing the expression of VHL and p53 (Kim et al., 2001). These proteins mediated HIF-α degradation. HDACi activated transcription of VHL and p53. Moreover acetylation of Lys532 of HIF-α stimulated its interaction with ubiquitination by VHL (Jeong et al., 2002). TSA and FK228 induce HIF-α degradation in VHL-null RCC4 cells (Demidenko et al., 2005) as well as Caki, Hep3B, DU145, PC3, U87, BT20, MCF7, and particularly, VHL^-/-^ cells such as RCC4 and C2 (Liang et al., 2006).

Bioinformatics was used to identify molecular mechanism of PT. COMPARE and hierarchical cluster analyses showed important cellular processes determining the sensitivity or resistance of tumor cells toward PT. Furthermore, we wanted to investigate, that NF-κB, HIF and their related networks are relevant to other cancer cell lines and not only MDA-MB-231-BCRP.

We subjected the data obtained from COMPARE analysis to IPA. Interestingly, IPA indeed revealed that many of the identified genes were related to NF-κB and HIF signaling networks. This further strengthens the importance of NF-κB and HIF as mechanism to explain the cytotoxic activity of PT against cancer cells. This result also fits together with the fact that we found many apoptotic genes, which are regulated by NF-κB and HIF. Furthermore, PT downregulated the expression of protein involved in glucose metabolism, angiogenesis, development and survival, all of which are regulated by HIF-α (Kim et al., 2017). Not surprisingly, carbohydrate metabolism appeared as one of the top signaling pathways using IPA analysis. The analysis further showed important cellular mechanisms such as cell cycle, cell death, cellular movement, cancer and other signaling pathways, which are directly or indirectly connected to NF-κB and HIF pathways. In addition, other pathways appeared illustrating the complexity of cellular responses to PT.

**FIGURE 12:**
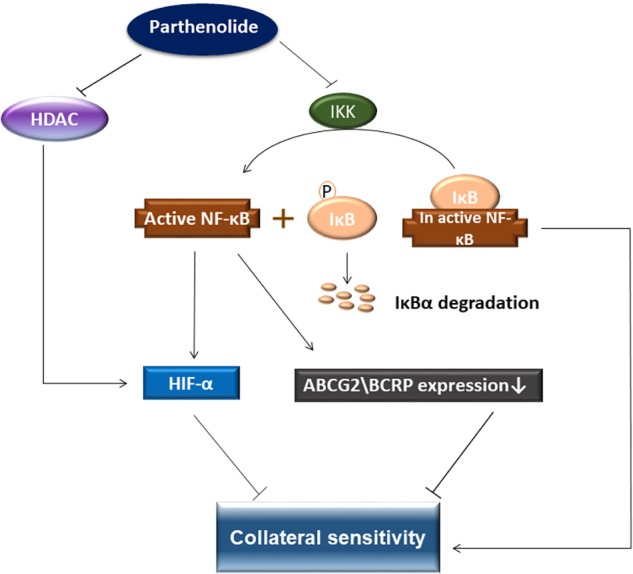
Schematic diagram presenting collateral sensitivity induced by parthenolide. BRCP transfected cells showed shows collateral sensitivity toward parthenolide by IKK inhibition, which prevented IκBα degradation and inhibited NF-κB. In addition, parthenolide also inhibited HDAC activity. HDAC stabilizes HIF-α. Decreasing the HIF-1α level activates apoptosis signaling pathways. NF-κB inhibition leads to downregulation of BCRP and HIF-1α and eventually to collateral sensitivity.

In conclusion, PT was active against various sensitive and drug-resistant cancer cell lines. In particular, BCRP-overexpressing MDA-MB-231-BCRP were collateral sensitive toward PT. Further investigations were carried out to understand the mechanism of collateral sensitivity (Figure 12). PT as known NF-κB inhibitor possesses also HDAC inhibitory activity, which both leads to inhibition of HIF-α. This phenomenon is suggested as mechanism of collateral sensitivity. COMPARE and cluster analyses predicted the sensitivity or resistance of cancer cells to PT using 47 NCI cell lines. Using a pathway analysis approach, we identified cellular functions and canonical pathways of genes involved in the mechanisms of action of PT.

## Author Contributions

MD carried out the cytotoxicity experiments, COMPARE and hierarchical cluster analyses, molecular docking, NF-KB reporter assay, IPA analysis, and Western blot and drafted the manuscript. EO performed the COMPARE and hierarchical cluster analyses. TE corrected the manuscript and supervised the project.

## Conflict of Interest Statement

The authors declare that the research was conducted in the absence of any commercial or financial relationships that could be construed as a potential conflict of interest.

## Footnotes

1 https://discover.nci.nih.gov/cimminer/oneMatrix.do

2 http://www.rcsb.org/pdb

## Abbreviations

ABC: ATP-binding cassette
BCRP: breast cancer resistance protein
EGFR: epidermal growth factor receptor
FBS: fetal bovine serum
HAT: histone acetylation
HDAC: histone deacetylase
PT: parthenolide
TP53: tumor suppressor gene 53
